# Patterns of morphology in children’s print: estimates of polymorphemic vocabulary using an innovative, AI-informed database of British and American word families

**DOI:** 10.3389/fpsyg.2026.1985734

**Published:** 2026-10-19

**Authors:** Clarence Green, Elfrieda H. Hiebert, Averil Coxhead

**Affiliations:** 1 Faculty of Education, The University of Hong Kong, Hong Kong, Hong Kong SAR, China; 2 TextProject, Santa Cruz, CA, United States; 3 Faculty of Humanities and Social Sciences, Victoria University of Wellington, Wellington, New Zealand

**Keywords:** artificial intelligence, corpus-based resources, K–12 literacy, morphological awareness, morphological databases, vocabulary instruction, word families

## Abstract

**Background:**

Morphological complexity is a pervasive feature of the vocabulary children encounter in print, yet resources for investigating morphology remain limited. What morpheme patterns do children encounter in print? Which morphemes cover how many polymorphic words in reading experiences? In this study we introduce the British and American Word Families (BAWF) project, an innovative open database of ∼378,000 words, and use it to address questions such as these.

**Methods:**

GPT-4.1 was fine-tuned using ∼6000 morphologically-coded words from an existing human-generated database (Morpholex). The fine-tuned LLM coded word-parts in all Wiktionary words attested in large British and American corpora. Performance was evaluated via agreement with existing databases. Classical roots were attributed to a subset of words by mapping a list of roots from Wikipedia to all related entries in Wiktionary. The resulting BAWF database is then used to estimate morpheme patterns across databases of children’s texts and further developed as an online application.

**Results:**

LLM word part predictions are good and fall within the range of agreement typical of existing databases. Nonetheless, output contains some error/noise. In children’s print, polymorphemic words increase across picturebooks to juvenile fiction and school-reading programs. Greek-Latin root words occur early and commonly. Estimates include that ∼100 prefixes/suffixes may account for nearly all derivations. The BAWF itself is released open-source https://cgg-projects.github.io/BAWF_V1/, with many lexical, morphological, and psycholinguistic variables, including but not limited to frequency, family size, and morpheme-counts.

**Discussion:**

The contribution suggests that instruction that prioritizes high-yield morphemes could give children access to large word families. These morphemes are present and common early in print. The BAWF (V1) helps see which may be useful to teach. Users can generate custom wordlists, targeting particular features and families, and access linked dictionary/etymological information. The platform includes AI-assisted searches, explanations, parsing review/corrections, and instructional support. The BAWF project will develop over time, incorporating additional variables (e.g., morphological transparency), improving accuracy and the user interface.

## Introduction

Proficiency in reading English requires an understanding of morphology, the system by which meaningful units combine to form words. Morphology in English is complex, inheriting three morphological systems: Germanic compounding (*blackboard, breakthrough*), Romance affixation through prefixes and suffixes (*disorder, complexity*), and Greek–Latin root morphology (*synthesis, biology*; Crosson et al., 2019; Hiebert, 2019). Robust relationships between morphological awareness and reading outcomes have been documented across diverse learner populations (Crosson et al., 2025; Deacon and Levesque, 2024; Korochkina and Rastle, 2025). Children who do not develop proficiency with English morphology face significant barriers to vocabulary growth and reading comprehension.

Classroom intervention research has also indicated value in morphological instruction, though how best to deliver this is ongoing research. While a growing body of research has produced sophisticated pedagogies (e.g., Bowers and Bowers, 2018; Lawrence et al., 2025; McKeown et al., 2018), systematic morphological instruction remains constrained by gaps in teacher preparation and the absence of accessible tools that make morphological relationships instructionally usable. The size, number, and depth of existing morphological resources restrict researchers’ ability to describe features of English that bear on reading development. These include the patterns of morphological complexity across children’s print (Korochkina and Rastle, 2025); the productivity of different morphological systems - compounding, affixation, the Greco-Latin morphology of academic vocabulary (Coxhead, 2000); and the relationships with word difficulty (Compton et al., 2024), instructional grade (Flor et al., 2024), and text frequency (Green et al., 2024).

This paper reports on the British-American Word Families (BAWF) project – a community resource of unprecedented scope^1^. We developed the BAWF using artificial intelligence, crowdsourced dictionary data, and state-of-the-art corpora of British and American English. It contains ∼378,000 words and estimates of their morphological properties. It can be used to identify and organize vocabulary by morpheme type, frequency, morphological system, and word family, among other dimensions. Our intention was to lower the technical and time barriers to morphological analysis.

In this study, we apply the BAWF to explore several patterns of morphology in children’s print experience, including how frequent polymorphemic words are, their developmental trajectories look like in their print materials, and estimate the most frequent Greek-Latin roots, prefixes and suffixes and the number of words they attach to in children’s print reading materials.

### The importance of morphological instruction

Morphological instruction is an active research domain, and its open questions motivate the BAWF project. In a recent meta-analysis, Colenbrander et al. (2024) examined 28 studies and 177 effect sizes, reporting small to moderate effects on reading and spelling, and concluded that morphology instruction should be part of literacy instruction. Nonetheless, the researchers point out that important questions remain, such as the optimal amount of instruction and which morphemes to teach. The optimal age is also unsettled, though it seems likely a good approach is a shift in emphasis from phonics to morphology in the primary grades (Korochkina and Rastle, 2025). For example, the English National Curriculum in the UK proscribes that instruction take place for a set of inflections (e.g., -s, -es, -ing,-d, ed) and derivations (e.g.,-er) by grade 1 (ages 5–6).

Morphological reading research has proceeded largely without a principled resource to estimate the morphological demands children face at different grade levels. Connecting intervention outcomes to the morphological properties of texts - family size, system membership, root productivity, frequency band - is analyses the BAWF project is designed to support. The BAWF project also addresses practical barriers: when preparation requires multiple resource consultations or specialized technical knowledge, adoption rates decline sharply (Desimone, 2009). A resource that consolidates frequency, morphological relationships, etymological origin, and system membership in a single resource reduces that burden.

### Morphological complexity in children’s print environments

Amongst the questions the project aims to shed light on is the patterns of morphological complexity in children’s print environments across the school years. How much morphological complexity do children actually encounter, how does it change across grade levels and text types, and which morphological systems account for that complexity? This bears on developmental accounts of vocabulary growth, the design of studies examining morphological instruction, and the construction of text difficulty models. For teachers and curriculum designers, it supports decisions about when, what and to whom to teach.

Nagy and Anderson (1984) estimated that approximately 60% of unfamiliar words students encounter in grades 3–9 are derivational words whose meanings can potentially be inferred through morphemic analysis. That estimate, foundational as it has been, was derived from a single frequency dictionary (Carroll et al., 1971) covering grades 3–9 by necessity rather than design, using methods that could not distinguish morphological systems, did not extend to the full school vocabulary, and could not attribute words to specific etymological families. Carlisle (2010) found that even in first grade approximately 25% of words contain more than one morpheme, rising to 30% by third grade, but these estimates too were bounded by the databases available.

Korochkina and Rastle (2025) recently advanced these estimates using MorphoLex (Sánchez-Gutiérrez et al., 2018) to estimate morphological information in an age-graded corpus representing children’s independent reading (the CYP-Lex) (Korochkina et al., 2024). They found that suffixation is the most productive mechanism for generating complex words, that complex words made up roughly half of distinct vocabulary and increased across age bands, and that many complex words occur at low frequency with poor distribution across books. However, MorphoLex’s coverage restricted automated coding to only 54% of distinct words in the corpus. A larger database would enhance our ability to estimate patterns in the school-aged reading lexicon.

The same constraint appears in Green et al. (2024), who compared morphological complexity in picturebooks and child-directed speech, finding that picturebooks expose children to richer morphological input than conversation, with higher rates of inflected words, morphemes per lemma, and derivational affixes. Yet, estimates relied on MorphoLex, limiting estimates of morpheme productivity. Similarly, the work of Dawson et al. (2023) examined morphological complexity across the Oxford Children’s Corpus (ages 5–14). This study found that morphologically complex words increased with audience age and were denser in nonfiction than fiction. Additionally, suffix distributions shifted from Germanic-origin morphemes in texts for younger readers toward Latinate in texts for older readers. Their analysis depended on MorphoLex, and a further 26% of the data required hand coding. Costs in time and resources are important to publicly funded research, and this limits the kinds of projects undertaken. These previous projects led us to think about how we might leverage LLM technology to help develop a more comprehensive resource.

The present study uses the BAWF’s expanded coverage to revisit and extend current estimates. An additional question within this broader research on morphological complexity is the structure and distribution of Greek–Latin morphology specifically. Academic and disciplinary vocabulary is disproportionately Greek–Latin in origin, and understanding when it first appears in children’s texts - and which roots carry the greatest coverage across the school vocabulary - has implications for both developmental research and instructional sequencing (Lane et al., 2019). Nagy and Townsend (2012) estimated ∼76% of academic words share common morphological roots and more than 90% of discipline-specific words are traceable to Greek or Latin. Crosson et al. (2019) demonstrated that knowledge of Greek–Latin roots facilitated reading comprehension even after controlling for vocabulary breadth, word reading fluency, and phonological awareness.

The practical value of bound root list for teachers is illustrated by Wei and Nation (2013), who identified 25 high-utility roots in the BNC-COCA lists - including *spec(t)-* (“look”), -*posit-* (“put”), *-vers-* (“turn”), and *-gen*- (“produce”)*.* They estimated these accessed approximately 300 mid-frequency word families, with nearly 100 additional stems each appearing in at least five mid-frequency words. Yet no existing resource has provided corpus-based estimates of when Greek–Latin morphology might begin to appear at scale in children’s print environments, the patterns across grades, or which roots carry the greatest coverage of vocabulary. The present study provides such estimates.

### Affix productivity and concentration across the school vocabulary

A second important question this project can help investigate concerns the productivity of English affixes across print environments: which prefixes and suffixes account for the greatest proportion of morphologically complex words children encounter, and how does that shift across grade levels? This matters to models of morphological development and to understanding which aspects of the affix system children are most likely to acquire through reading exposure alone. For teachers, it bears on instructional priority - identifying the affixes that provide the greatest leverage.

The theoretical grounding comes from Nagy and Anderson’s (1984) observation that morphological knowledge has multiplicative value: knowing one base or affix potentially unlocks multiple related words. White et al. (1989) gave that observation empirical shape, finding that 20 prefixes account for nearly 97% of prefixed words most frequently appearing in school texts, and that the top 10 suffixes account for 85% of suffixed words. Those figures have shaped curriculum and instruction for decades. They were derived, however, from a single frequency dictionary using methods that predate modern corpus linguistics, and they measured frequency of encounter rather than generative reach - the number of distinct wordforms an affix attaches to. These are not equivalent: an affix that appears often may attach to few bases, while one that attaches to many bases gives a reader broad inferential leverage over unfamiliar words regardless of the frequency of any individual derived form. A database with the scope and structure of the BAWF makes it possible to reexamine these estimates at different points of schooling.

### Design principles for a new morphological database

Designing a morphological database for K–12 education and research requires clarity about which features matter and why. Three features are particularly valuable: (a) frequency information, enabling users to search, sort, and filter words and morphemes by how commonly they appear in text; (b) morphological relationships, enabling users to identify and explore word families that share base words, roots, or allomorphs; and (c) system-based organization, enabling users to locate and compare words by morphological system-Germanic compounding, Romance affixation, and Greek–Latin root morphology. Together, these features support both the empirical description and measurement that reading research requires and the instructional planning that practitioners need.

#### The importance of frequency-based information

Frequency annotation is a prerequisite for describing the distribution of morphological complexity across the lexicon. For example, Hiebert et al. (2024), analysing 300,000 words sampled from 12,000 digitized U.S. school texts, found that 76% of rare words - those appearing less than once per million words - were morphologically related to higher-frequency word families. This suggested that morphological knowledge extends the reach of frequency-based instruction well into the low-frequency range. Findings of this kind, however, have depended on purpose-built analyses of specific corpora. What has been missing is a general-purpose resource linking morphological structure to frequency across the English lexicon.

A database enabling estimation of the prevalence and productivity of different word family types across the full frequency range is essential for characterizing the morphological demands of print (Bauer and Nation, 2020; Carlisle, 2010). For instruction, frequency annotation supports principled decisions about which morphological families to teach explicitly and which students are likely to acquire incidentally through reading. Generally speaking, because higher frequency word families cover more of the words in text, curriculum should typically progress from more to less frequent vocabulary.

#### The importance of word family information

A word family is a set of morphologically related words (Brysbaert et al., 2016), though operationalizations vary across vocabulary research in education, cognitive science, linguistics, and applied linguistics. One important distinction in understanding word families is the difference between a root and a base. A root is the irreducible core of a word - the morpheme remaining after all affixes are removed, which may be free or bound. A base is a standalone English word carrying core meaning and serving as the foundation for derived forms. Wordforms may be connected into a “family” by roots and/or bases. The word *additional*, for example, contains the base *addition* and the root *add*; *spectator* contains the base *spectate* and the bound root *spec(t*); *runner* has *run* as both base and root (Aronoff and Fudeman, 2022; Bauer and Nation, 2020; Haspelmath and Sims, 2013).

Word families can also be operationalized as sets sharing a bound etymological root: *spectator–spectacular–aspect*. Teaching productive bound roots such as *spect-, struct-, port-*, and *tract-* has become increasingly common in K–12 instruction (Mesmer, 2024), yet estimates of which etymological roots carry the greatest coverage across the school vocabulary - and at what grade levels they first appear at scale - have not been available (though see Lane et al., 2019, for a study of ∼ 4,907 academic words). A database enabling searching and sorting by etymological root, with prevalence and productivity data across K–12 text corpora, would fill a significant gap for both research and instructional design.

Word family structure is itself a research variable of considerable scope. Brysbaert et al. (2016) showed that approximately 18,269 base words account for over 65,000 lemmas - each lemma representing a distinct inflected or derived form - establishing family membership as a fundamental structural property of the English lexicon. Grouping words by family makes it possible to estimate how much vocabulary in any corpus is morphologically related, to examine how family size interacts with frequency and text difficulty (Webb and Nation, 2017), and to investigate how family type distribution shifts across grade-level texts. Nagy and Anderson (1984), analysing 7,260 words from a single frequency dictionary (Carroll et al., 1971), estimated that for every word children learn they may be able to infer three to four additional derived forms - a finding whose influence on the field has been substantial but whose empirical basis was necessarily constrained by the limited resources of its time. A database that makes family structure searchable and quantifiable across the full school lexicon makes it possible to revisit that estimate with an instrument commensurate with the question.

#### The importance of system information

English morphology inherits its word-formation patterns from three major historical systems-Germanic, Romance, and Greek–Latin, each with distinct structural properties. Germanic compounding combines typically short, concrete morphemes, such as *afternoon (after + noon*) and *blackboard (black + board)*. Because compound meaning is often compositional, students can frequently infer unfamiliar forms from their parts, making compounding a productive entry point for vocabulary instruction.

Romance affixation operates through prefixes and suffixes attached to Latin-origin roots: pre-*view, im*-possible*.* This system grows in prominence across the school years: Korochkina and Rastle (2025) documented a developmental trend toward greater affixational complexity in age-aligned texts, contributing to the vocabulary demands students face as they advance. Greek–Latin morphology dominates academic and technical registers-*photo-synthesis* and *geo-graphy*. Unlike Germanic compounds, classical roots tend to be less transparent and, unlike Romance affixes, they combine in multiple configurations. They are, however, a more central morphological resource in academic vocabulary (Coxhead, 2000; Crosson et al., 2019).

A large-scale resource organized by morphological system would enable two contributions that current databases have not been designed to support. First, it would make possible empirical estimates of the prevalence and productivity of each system across the full range of vocabulary children encounter in print, disaggregated by grade level and text type: How much of the vocabulary load in early-grade texts is Germanic? When does Romance affixation become numerically significant? At what grade level does Greek–Latin morphology begin to carry the greatest weight? Second, system-based organization would allow researchers and curriculum designers to identify words sharing specific morphological properties - a particular affix, compounding pattern, or classical root - enabling principled analysis of text demands and more evidence-grounded instructional sequencing.

### Available resources for research and practice

Existing resources fall into four categories, each offering value but partial utility and leaving important needs unmet. Professional books and teacher-facing texts such as *Unlocking Literac*y (Henry, 2003), *Speech to Print* (Moats and Brady, 2020), and *Words Their Way* (Bear et al., 2020) provide valuable morphological frameworks and structured word lists. But a teacher/researcher seeking all derivations of, for example, *view* or all words sharing the prefix *pre-* must reconstruct that information piecemeal; no single searchable interface consolidates morphological relationships, frequency, and etymological origin in one place. Online resources such as Wiktionary and etymonline.com answer individual word questions but cannot compile, sort, or filter morphological word families for research or lesson planning.

Frequency-based word-family lists, most notably Nation’s (2016) BNC-COCA lists, have been invaluable for applied linguistics and L2 instruction. Their frequency-ranked organization, however, creates challenge for K–12 morphological work: words sharing the same morphological root are routinely separated across lists by frequency. *View* and *preview* share the prefix *pre-* and the root *view*, but because *preview* is far less frequent than *view*, they appear in different lists entirely. A teacher or researcher trying to assemble morphologically related words across the school vocabulary cannot rely on these lists to do so. Furthermore, the BNC-COCA lists largely exclude the Greek-Latin morphology that becomes central to academic and technical vocabulary in the upper elementary grades and beyond - vocabulary that carries substantial weight in text comprehension and content-area learning.

The Academic Word List (Coxhead, 2000) and Academic Vocabulary List (Gardner and Davies, 2014) partially address this gap by capturing vocabulary that is disproportionately Latinate and Greek in origin. The AWL, drawn from a 3.5-million-word corpus of academic prose, identifies 570 word families accounting for approximately 10% of academic text tokens. The AVL, derived from a substantially larger 120-million-word academic subcorpus of COCA, identifies 3,015 lemmas covering approximately 14% of academic text. Both resources have been widely used in vocabulary research and instruction, and both have meaningfully advanced understanding of academic language demands. Neither resource was designed to include fine-grained morphological information of the academic words, nor answer the question of how much and what kind of morphological complexity children encounter in actual school texts across the K–12 span.

Among research-focused databases, MorphyNet (Batsuren et al., 2021) is the largest existing morphological database at approximately 225,000 derived words linked to their base forms, but it was developed for natural language processing rather than for educational research or instruction. Its structure is a list of baseword plus one derivation per line, with no grouping by family, root, or affix, and no frequency information. This limits its utility for studying English morphology in K–12 contexts and makes it difficult for researchers without computational expertise to use.

MorphoLex (Sánchez-Gutiérrez et al., 2018) is the most psycholinguistically rigorous database available, containing both root- and base-word information alongside morphological segmentation for approximately 70,000 words, and has been used productively for estimating morphological features of children’s print exposure (Korochkina and Rastle, 2025). It derived mostly, in turn, from the parses in the English Lexicon Project (Balota et al., 2007). Its coverage ceiling, however, constrains what researchers can estimate: words outside its ∼70,000-word scope are simply unanalyzable. It also offers no practical way to search, sort, or filter by root, base word, or affix, and no frequency information to distinguish more from less common word families. Despite its value, these limitations restrict its utility for both large-scale corpus research and instructional applications. Overall, the limitations of existing resources have defined the boundaries of what researchers and teachers have been able to investigate.

### The present study

Furthering the research reviewed above requires data on the morphological properties of English. To that end, we undertook the BAWF project to develop a database using an innovative AI approach. The three research questions that follow reflect what can be directly addressable by such a database in terms of characterizing children’s print environments and informing instructional decision-making.

RQ1. In print that children are exposed across school years, how much vocabulary is estimated to be morphologically complex based on the BAWF?

RQ2. How much of this vocabulary is estimated to contain Greek–Latin roots?

RQ3. Using estimates based on the BAWF, what are the most productive prefixes and suffixes, and what is their lexical coverage?

In RQ1, we use the BAWF to estimate morphological complexity in English and samples of children’s print environments, drawing on four newly available lexical databases. Additionally, we estimate in RQ2 the prevalence of morphologically complex words and Greek–Latin roots in this data. Finally, in RQ3, we use the BAWF to estimate which prefixes and suffixes are the most productive, which we define as the number of wordforms they attach to.

Within the methods section, we also report on the scope and accuracy of the morphological information in the BAWF database compared to existing databases. The BAWF (V1) parses are not intended to represent a theory of morphological structure, representation or processing, whether morpho-orthographic decomposition, dual-route models, or connectionist accounts. Relatedly, the contribution is restricted to English, so other languages need to be the object of future research and development. Rather, the aim is to develop a broad-coverage morphologically-annotated dataset, with a reasonable signal-to-noise ratio, and thereby extend prior similar resources such as MorphoLex (Sánchez-Gutiérrez et al., 2018) and the English Lexicon Project (Balota et al., 2007).

## Materials and methods

### Lexical data

Two types of lexical data were used in this study. The first comprised the source data used to develop the BAWF itself and the second consisted of four newly available lexical databases used to establish the viability of the BAWF as a measure of morphological complexity in early print vocabulary.

### Source data

We downloaded the entire open-source Wiktionary dictionary^2^ of ∼8.7 million words in machine-readable (JSON) format^3^ (Ylonen, 2022). To ensure only words used in contemporary English were included in the BAWF, we filtered Wiktionary entries the words in the ∼100-million-word British National Corpus 2014 (BNC_2014; Love et al., 2017) and the over 1-billion-word Corpus of Contemporary American English (COCA; Davies, 2019). Both are the state-of-the-art corpora representing their respective national varieties (Brezina et al., 2021). Only words attested in at least one of the two corpora and had a Wiktionary entry were retained.

This resulted in approximately 378,000 correctly spelled words for further processing by generative AI. The BAWF also contains frequency information from the BNC_2014 and COCA, namely computing log10 (frequency per billion words) for each corpus and averaging the log (when a word occurred in only one corpus, only that frequency was used). The frequency-information unit is called the Zipf (van Heuven et al., 2014) and ranges on a Likert scale from 1 to 7 (least to most frequent). A Zipf value of 1 is approximately 1 occurrence per 100 million words; a value of 2 represents once per 10 million words; and a value of 3 represents once 1 per million words. Raw corpus counts for individual corpora are not listed in accordance with COCA’s terms of use, but they can be obtained from https://www.english-corpora.org/.

### Criterion databases

To address the second research question regarding children’s print experience, we identified several recent databases: (a) the Children’s Picture Books Lexicon (CPB-Lex; Green et al., 2024), a lexical database derived from children’s picture books providing frequency norms for approximately 25,585 word types; (b) the Vocabulary eXpected Grade Levels (VxGL; Flor et al., 2024), a vocabulary list that assigns words to U.S. school grade levels; (c) the Developmental English Lexicon Project (d-ELP; Compton et al., 2024), a database containing reading difficulty and grade estimates for 9,961 frequently printed English words, based on data collected from 1,907 first to fifth graders; and (d) the Children and Young People’s Books Lexicon (CYP-Lex; Korochkina et al., 2024), derived from 1,200 popular books in the U.K. and stratified into three age bands (7–9, 10–12, and 13+ years); e) the juvenile fiction section of the Corpus of Contemporary American English (Davies, 2008); f) word-frequency data from the most popular core reading programs in the US, developed by Graves et al. (2019) and representing printed-words from grades 1–6.

### AI model fine-tuning

The LLM used in the study was GPT-4.1^4^. First, we wrote code to create a training file (json) used to “fine-tune” the LLM. Fine-tuning is a process of providing feedback (the “ground truth”) to an AI after it generates a prediction about some data, thereby optimizing it for a set task (Conde et al., 2025). We asked the LLM to code the morphological components of a word; feedback came from MorphoLex (Sánchez-Gutiérrez et al., 2018). The model adjusted its predictions closer to the ground truth across 1650 learning cycles, and this fine-tuned model was then saved. The training set was 6,000 words (≈ 8.5%) from MorphoLex: 1,000 compound words, 2,000 prefixed words, 2,000 suffixed words, and 1,000 nonderived simplex words. We chose MorphoLex as “ground truth” because it is the only existing database that separately codes roots, base words, affixes, and compounds, and it has shown relationships to word processing behavior and other psycholinguistic data (Sánchez-Gutiérrez et al., 2018). Yet, one of the limitations of MorphoLex as training data is that it does not code inflectional morphology. Inflectional morphemes matter to early lexical acquisition and decomposition. Future versioning in the BAWF project may include parses with non-derivational morphemes (Hiebert, 2019).

#### AI model deployment

We deployed the fine-tuned GPT-4.1 to code the morphology of the 378,000 target words. We first gave the AI a system prompt that explained the task, specified its role as an expert on English morphology, and constrained the output to a structured JSON parse. In the main prompt (see Figure 1), we provided the LLM with the definitions of a root, base word, prefix, and suffix, and then gave it coding instructions matching those used in MorphoLex. Few-shot prompting was used to enhance performance (i.e., examples of words+coding were embedded in the prompt, *n* = 15). We accessed the AI via an application programming interface and Python code. The complete code is provided in the Open Science Framework (OSF), and the code snippet in Figure 1 is the main prompt.

**FIGURE 1:**
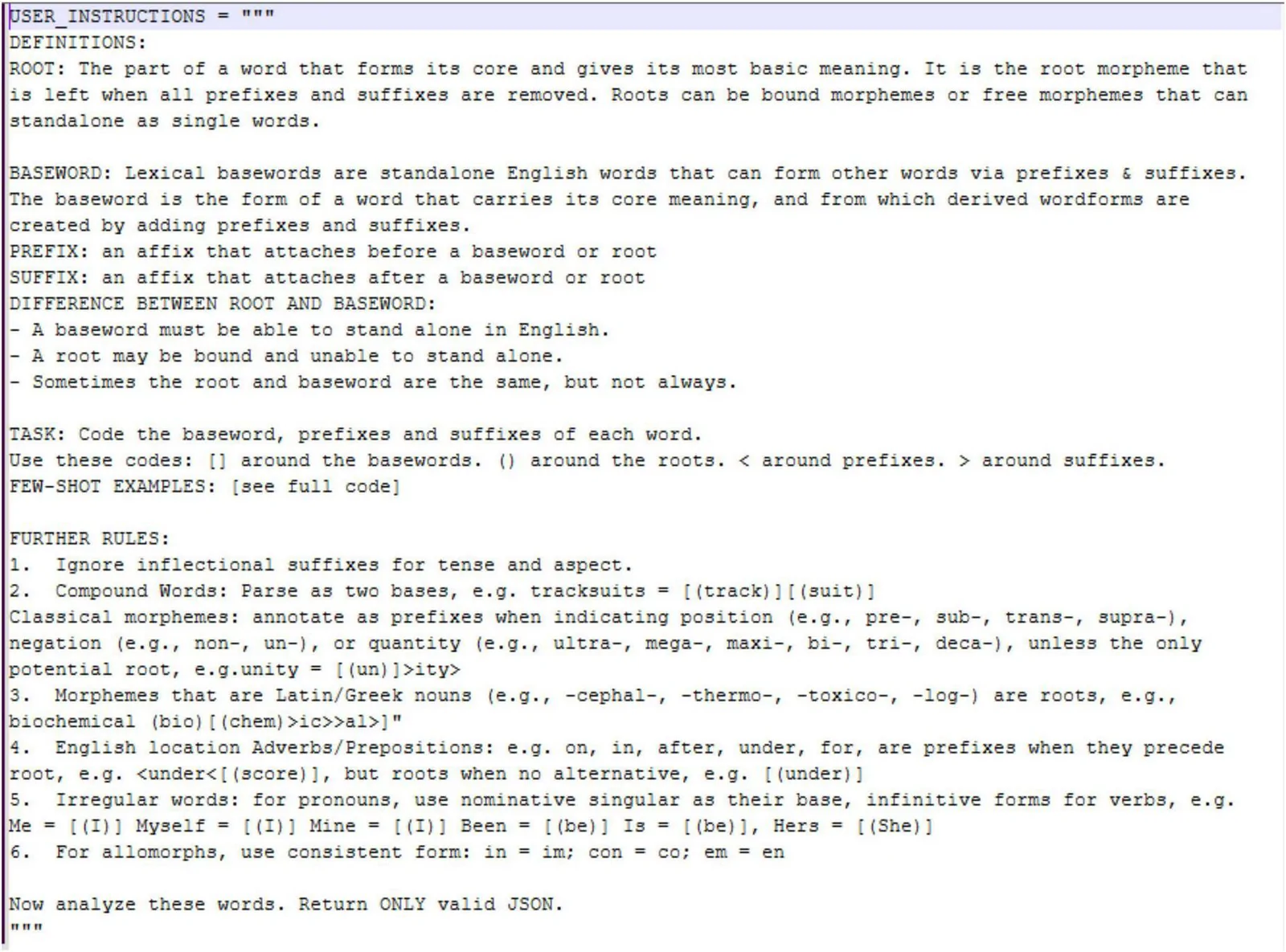
Code snippet with prompt given to GPT 4.1.

The temperature setting of the model was 0, which generates more deterministic, reproducible output (Conde et al., 2025). We processed the master list in batches of 50 words to balance financial costs with LLMs being affected by contexts. That is, other words in the input at the same time can affect predictions (Brysbaert et al., 2025: Conde et al., 2025). The output was the list of words morphologically coded with MorphoLex conventions: word roots between (), base words between [], prefixes segmented with <, and suffixes with>. Examples include unattractive <un<[a<(tract)]>ive>’ extracted [<ex<(tract)]; construe [<co<(strue)]; showroom [(show)][(room)]; and causality [(cause)]>al>>ity>. Following the original coding scheme, there is some normalization of allomorphs, such as con/co, im/in, and the model was instructed at deployment and fine-tuning to follow MorphoLex and not code inflections.

#### Adding etymological roots from Wiktionary

Wiktionary was the source of classical root etymology information. First, we wrote code to extract crowdsourced lists of common etymological roots listed in Wikipedia^5^. This list contains 1,693 different Greek–Latin root morphemes and their allomorphs, illustrated by 14,462 example words. We wrote code to extract from Wiktionary these 14,462 words and all other words under the “Derived Words” and “Related Words” subheadings of their dictionary entries, which list words with the same root. After removing multiword expressions, this gave 81,617 word forms. We then searched for matches between these Wiktionary words and any words, lemmas, bases or roots in the BAWF. When matches were found, the entry had the suggested Greek–Latin root appended to a new column alongside it. We further checked that BAWF Wiktionary entries mentioned a classical etymology and removed suggested roots when not found.

Wiktionary lists prefixes^6^, which also contains allomorphs and meanings, so these were extracted and aligned with words in the BAWF that contained matching prefixes (based on the GPT parse). Wiktionary suffix information^7^ unfortunately lacks meanings and allomorphs. To add any missing prefixes or suffixes that the LLM may have missed we crawled Wiktionary’s list of 139,058 prefixed words^8^ and 166,072 suffixed words^9^. If a word in BAWF occurred in these Wiktionary data with an affix but had an empty affix slot after the GPT parse (this was relatively uncommon), the missing prefix or suffix was added.

### Database creation and additional variables

To create the database (see text footnote 1), the AI output was formatted into a spreadsheet with the word form in the first column, followed by the base word, root, prefixes, and suffixes. Compounds were placed in a separate spreadsheet to the main database, with added columns for multiple roots and bases. We computed frequency and family size, both for roots (the number of word forms sharing the same root) and base words (the number of word forms sharing the same base). The main database has two estimates of family size and word family frequencies: one with compounds included and one without. In the compound words spreadsheet, family sizes had to be computed slightly differently, so there for each compound, its family size and family frequency is the number of words in the main database *and* the compound spreadsheet that contained *any* of the bases (base word family size) or roots (root family size) in the compound word.

In the BAWF, prefixes and suffixes in a word form are root affixed, not base-word affixes, following MorphoLex. In the compound spreadsheet, for each root the prefixes and suffixes are computed as prefix 1 on root 1, prefix 2 on root 1, prefix 1 on root 2 (if any), and so on. Columns were added with estimated familiarity (how well-known a word is), age of acquisition (AoA) and grade level appropriateness. The familiarity estimates derive from Brysbaert et al. (2025), who used GPT 4o to rate approximately 400,000 words/expressions on a Likert 1–7 scale for familiarity, prompting the AI with the following: “A word is very FAMILIAR if you see/hear it often and it is easily recognizable. In contrast, a word is very UNFAMILIAR if you rarely see/hear it and it is relatively unrecognizable” (p. 27). These ratings were validated against human ratings. This variable allows users to filter for well-known words. Brysbaert et al. (2025) suggested more than 90% of people tend to know words rated 3.5 or above.

While we have argued above of the value-added of an extensive database, we also recognize that for teachers, low frequency and unfamiliar words will be less used. Therefore, we additionally created a curated set of word families in the main webpage. The focused database is a trimmed version that lists only word families with familiarity ratings above approximately 3.5, thus words that are widely known by most speakers of English (∼90%), following Brysbaert et al. (2025). These words are likely to be more useful for teaching than words that are not well known by English-language speakers.

The AoA estimates derive from Green et al. (2025), who crowdsourced estimates for reading AoA for 11,074 early acquired words (listed < age 10 in Kuperman et al., 2012) with the following prompt: “When you are estimating your reading acquisition of a word, we mean the age at which you would have understood that word if you read it in print” (p. 303). Estimated grade-level appropriateness derives from the Vocabulary eXpected Grade Levels (VxGL: Flor et al., 2024) which assigns words to U.S. grade levels from 0 (kindergarten) to 16 (fourth year of college). It was developed from a corpus of instructional wordlists used by teachers and extended to over 120,000 words via regression modelling.

The online BAWF project page is an interactive, evolving resource for researchers and educators. Users can search, filter, sort, download, and generate customized wordlists based on lexical, morphological, and psycholinguistic variables (e.g., word frequency, family size, base word, root, affix, compound status, and morpheme count). To support classroom applications, features include interactive word-cloud visualizations and an AI prompt generator to translate data into instructional materials.

Each word entry includes “Action” buttons, with links to dictionary and etymological resources, AI-assisted explanations and LLM-supported morpheme-parse reviews. These functions are intended to help users interpret a parse, understand the meanings of word parts, and evaluate possible alternatives. These are important dynamic checks-and-balances because BAWF (1.0) is not perfect in every entry. As with other large-scale lexical resources, such as the BNC/COCA word-family lists (Nation, 2017), the BAWF will improve through successive updates.

We created the AI Morphology Studio tab, which is an AI-powered search interface for the BAWF where users can ask the BAWF questions though an LLM. It allows users to search the database using natural-language classroom or research requests, such as “find me familiar words with the prefix un-” or “show all grade 4 words with the root spect.” It then returns matching word-family records, explains the search in accessible language, and supports the creation of instructional word sets, prompts, word clouds, and classroom activities.

Releasing a fully accurate static set of morphological codes for hundreds of thousands of words is currently limited by theoretical ambiguity in morphology, human and financial resource constraints, and the practical and environmental costs of large-scale LLM use. Instead, BAWF is designed as a transparent, useful, and revisable resource that supports both large-scale analysis and word-level inspection. For example, with disliked coded as dis+like, the tool explains that this parse reflects the derivational prefix dis- and base like, while noting that the inflectional ending -ed is part of the wordform. Users can submit corrections, feedback, and alternative analyses for individual entries, which will inform future updates and help BAWF develop as a community-supported resource for research, teaching, and curriculum development.

The platform’s “Review Parse” feature allows users to check the parse within the page using Llama 3.2 3B Instruct through Cloudflare Workers. We programmed the webpage to draw on this model because it is open source. In addition, users can access frontier models, by clicking on the “Explain” button, which links to Gemini, Claude or GPT, depending on user preference. A “Report” button functions as a crowdsourcing mechanism that can inform improvements to parses over time. All in all, there is more than enough information and cross checks in the database not to lead any teacher or user astray from possible parsing error.

The BAWF project (data, code) is available free under Creative Commons at the OSF^10^ and developed on Github (see text footnote 1), see Figure 2. The main database has 64+ variables in separate columns; Table 1 provides a summary.

**FIGURE 2:**
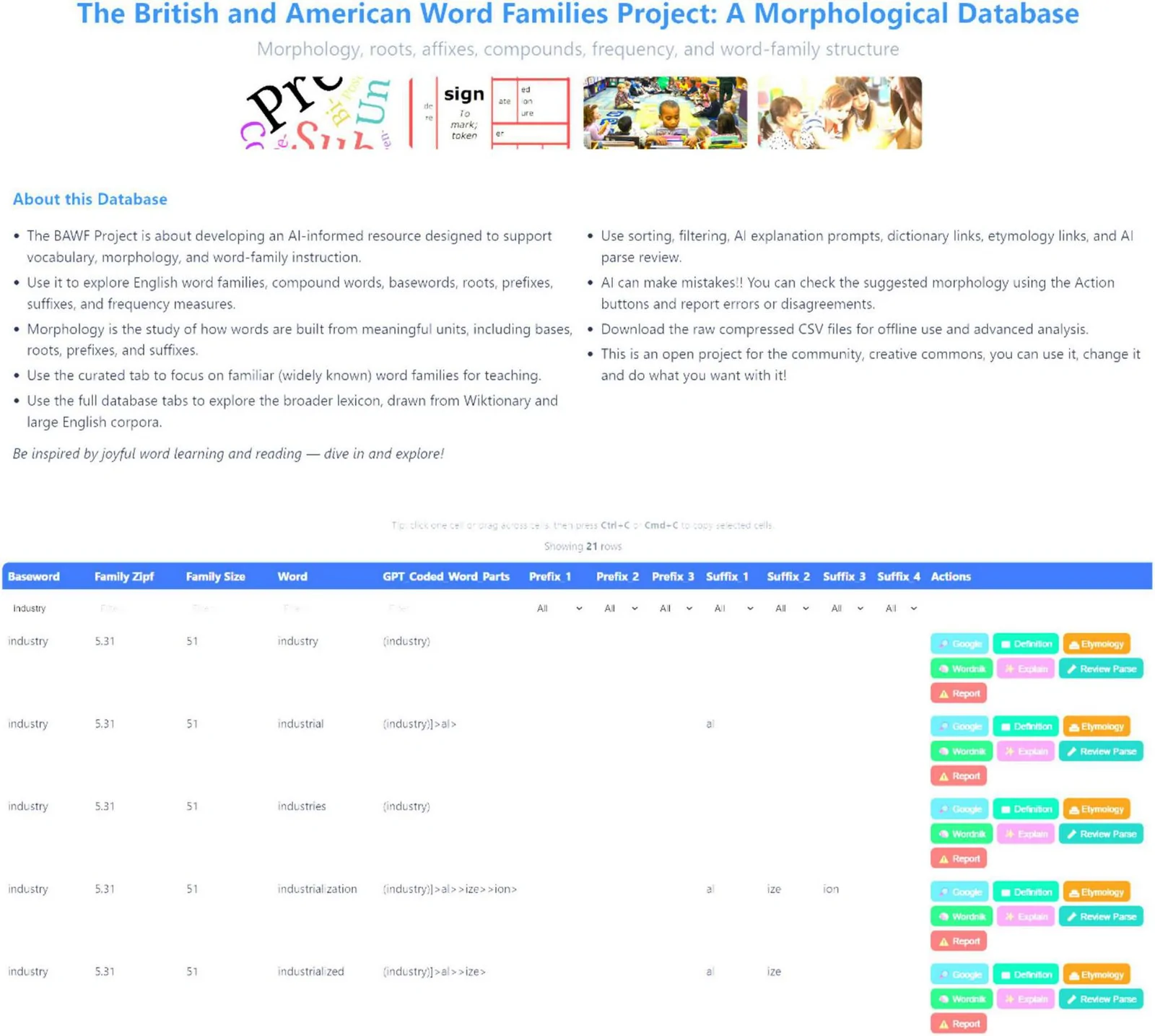
The BAWF project (Online) (https://cgg-projects.github.io/BAWF_V1/).

**TABLE 1:** Search, sort and filter columns in the BAWF.

Column name	What it means	Best used for
Word	The dictionary wordform	Finding a target word
Word_frq_Zipf	How common the word is	Sort by frequency
Baseword	GPT suggested lexical base	Group words by base
Root	GPT suggested root	Group words by root
Greek-Latin root	Greek-Latin roots (some > 1)	Group words by etymology
Greek-Latin meaning	Meaning of classical root	Filter for related meanings
Root (or) Baseword Family size	How many words with same base/root (+/- compounds)	Find large word families (columns also for freq.)
Prefix_1 (etc.,)	The first (outermost) prefix	Filter for specific prefixes
Suffix_1 (etc.,)	The first (outermost) suffix	Filter words with suffixes
N_Morphemes	Total morphemes in the word	Filter by word complexity
Reading AoA*	Reading age-of-acquisition	Readability / difficulty
VxGL*	Expected instructional grade	Educational level filtering
GPT_Familiarity*	How familiar the word is	Filter well-known words

*Reading AoA (Green et al., 2025), VxGL (Flor et al., 2024), GPT Familiarity (Brysbaert et al., 2025).

### Scope and accuracy of the BAWF database

The BAWF consists of approximately 378,000 words, separated into a main database of ∼320,000 words, a compound word database of ∼58,000 words, and a curated word family list of ∼16,000 familiar word families. The curated wordlist consists of basewords and derivations with familiarity ratings above 3.5 (Brysbaert et al., 2025), ordered by corpus frequency of the entire family. This is the largest morphology resource ever constructed, essentially all words in Wiktionary in use in the largest contemporary corpora of American and British English. In Table 2, the distributional information and coverage of the database is summarized.

**TABLE 2:** Scope of the BAWF.

Data	*N*
Wordforms (excl. compounds)	319,999
Compounds	57,945
Unique base words	163,781
Unique roots	157, 739
Unique Greek-Latin roots	1,437
Wordforms with Greek-Latin roots (excl. compounds)	76,749
Unique prefixes	1016
Prefixed wordforms	69,286
Unique suffixes	1125
Suffixed wordforms	94,448
Wordforms > 2 derivational morphemes (excl. compounds)	120,696
Average baseword family size	1.95
Average root family size	2.02
Average Greek-Latin root family size	54.58
Curated word families (shared basewords, familiar)	15,991
Curated word families average family size	3.14
Curated word families number of wordforms	50,237

Table 2 illustrates several immediate contributions of the BAWF as a basis for estimates about the English language and its demands on readers. One notable result is that ∼55% of word forms are morphologically complex (two or more derivational morphemes or compounds). The BAWF suggests that ∼69,286 words have prefixes, and ∼94,448 contain suffixes. Especially interesting is that even though there are only ∼1,437 unique Greek-Latin roots associated with the entries; they are extremely productive. On average, each root supports 54 different word forms, showing the extensive foundation classical roots provide for English vocabulary.

The LLM coding was evaluated by comparing it to ground truth from MorphoLex and cross-referencing with expected agreement levels of existing human-generated databases. The comparisons between GPT 4.1 and MorphoLex include the overall word-level coding. This means the overall percentage of words to which GPT gave the exact same coding as in MorphoLex. In addition, we checked agreement for basewords and roots. Comparisons amongst other established databases are restricted to basewords because they do not have root/coding information. Agreement is compared amongst the BNC-COCA lists, MorphyNet, MorphoLex, and the word family list from Brysbaert et al. (2021) - which represents words known to English speakers in a crowdsourcing megastudy.

Table 3 shows that GPT-4.1’s coding achieved levels of agreement comparable to those observed between existing human-constructed morphological databases. For the 65,900 words that appeared in both the GPT-4.1-coded BAWF and MorphoLex, agreement was 73%. Component-level agreement was higher: 79% for roots and 81% for basewords. Importantly, when words from the MorphoLex training data were excluded, these agreement levels were retained: ∼72% for overall coding, ∼82% for base words, ∼79% for roots. The estimated number of derivational morphemes in the BAWF also correlated strongly with MorphoLex morpheme counts, *r* = 0.80.

**TABLE 3:** Percentage agreements for GPT-4.1 and across morphological databases.

Comparisons: GPT 4.1. vs. MorphoLex (*n* = 65900)	Percentage agreement
GPT 4.1. vs. MorphoLex- overall coding	73
GPT 4.1. vs. MorphoLex: roots	79
GPT 4.1. vs. MorphoLex: basewords	81
Agreement levels of existing morphology resources [base words]	
MorphyNet base words vs. BNC-COCA lists (*n* = 27504)	50
MorphyNet vs. MorphoLex (*n* = 19978)	49
BNC-COCA lists vs. MorphoLex (*n* = 54280)	74
Brysbaert et al. (2021) vs. MorphoLex (*n* = 30338)*	79#
Brysbaert et al. (2021) vs. BNC-COCA lists (*n* = 38001)*	81#

*Excludes 381 bases in Brysbaert et al. (2021) marked # (formatting impeded comparisons).

Further, Table 3 shows that these figures fall comfortably within the range of agreement between established human-generated morphological resources for base words. Inter-resource agreement varies widely, from as low as 49%–50% (MorphyNet vs. MorphoLex; MorphyNet vs. BNC-COCA) to 74%–81% (BNC-COCA vs. MorphoLex; Brysbaert et al. vs. MorphoLex/BNC-COCA). GPT-4.1’s performance is on par with most existing resources.

While agreement does not mean accuracy for every individual word, especially because human-coded resources can contain debatable parses and error, nevertheless these results suggest that the fine-tuned GPT-4.1 produced a reasonably good outcome in terms of signal-to-noise for a database at this scale. Readers may be interested that we spent 2+ years and many resource costs exploring different methods before settling on the current one, but never could exceed the above agreement levels. We wonder if that is because these thresholds may be about the best that can be achieved with current LLM technology, and are excited about what future models may offer the BAWF project.

One point-of-view suggested to us was that disagreements among human-generated resources reflect principled theoretical differences, while disagreements with the LLM reflect automated parsing error. Looking at entries that do not match with Morpholex or the other databases’ basewords, one sees parsing errors yet also many cases where the LLM generates reasonable analyses. For example, *zoological* in Morpholex is [(zoo)(log) > ic > al > ] versus the LLM’s [(zoo)(log) > ic > ] > al. The former suggests *zoological* is a baseword in itself; the LLM suggests *zoologic* is the base suffixed with -*al*; the BNC-COCA lists *zoology* as the baseword and Brysbaert et al. (2016) suggest two possible word family bases: *zoo* and *logy.*

Grammatical indeterminacy is a well-established feature of linguistic analysis: the boundary between a transparent derived form, an opaque historical form, and an unanalyzable lexical item is often gradient rather than categorical (Aarts, 2007). Although some words have easily identifiable morphemes, many do not, and a morpheme may retain a meaning-form correspondence to varying degrees of salience (Haspelmath and Sims, 2013).

Previous psycholinguistic research suggests larger and more frequent morphological families facilitate word processing, as indexed by faster lexical decision times/LDT (Sánchez-Gutiérrez et al., 2018). We conducted hierarchical linear regressions from these BAWF variables on the English Crowdsourcing Project LDT data (matched wordforms *N* = 49,250, excluding compounds). BAWF wordform Zipf frequency and length were entered in Step 1, as these typically account for most LDT, and the baseword family frequency and family size entered in Step 2 and compared to see if they added any additional change in LDT.

Word length and frequency alone accounted for 56.4% of the variance. Adding baseword family frequency and family size significantly improved fit (ΔR^2^ = 0.048, *p* < 0.001). Standardized coefficients showed that baseword family frequency uniquely predicted faster response times (β = –0.267, *p* < 0.001), though baseword family size showed only a small positive association after length and surface wordform frequency s (β = 0.021, *p* < 0.001). The same pattern was observed for root measures (ΔR^2^ = 0.042, *p* < 0.001)- Root family frequency showed a unique facilitatory effect (β = –0.258, *p* < 0.001), while root family size showed a small positive coefficient (β = 0.044, *p* < 0.001). Variance inflation factors for all predictors were below 3, indicating that multicollinearity was not problematic.

Overall, these data suggest BAWF family frequency estimates add some explanatory power when regressed against LDT, though little effect for family size estimates when length and wordform frequency are controlled. One possible explanation is that, in a database of this scale, many families are very small because they are low frequency (e.g., hapax), which may limit the independent contribution of family size. Future research could examine family-size effects restricted to subsets, such as semantically transparent and/or higher-frequency families.

## Results

### RQ1: In print that children are exposed across school years, how much vocabulary is estimated to be morphologically complex based on the BAWF?

In Table 4, we report the coverage achieved by the BAWF across recent databases of words developed to support K–12 reading and instruction research, as well as the number of polymorphemic words. Estimates of complexity represent words in the BAWF with 2 or more morphemes, excluding inflections. To reduce noise, estimates exclude words from the target datasets not in Wiktionary.

**TABLE 4:** Estimated morphological complexity in early print vocabulary.

Database	N word types	BAWF coverage	Compounds N-%		Complex N-%	
CPB-Lex	25,564	83.50%	1,443	6.76%	6,384	29.91%
CYP-Lex 7–9	52,847	97.45%	5,313	10.32%	21,770	42.27%
CYP-Lex 10–12	70,941	95.84%	7,256	10.67%	29,318	43.12%
CYP-Lex 13+	90,976	94.79%	9,203	10.67%	39,498	45.80%
COCA Juv_Fic	53,033	80.13%	3,877	9.12%	15,965	37.57%
CRP-US grade 1	3,070	97.23%	163	5.46%	522	17.49%
CRP-US grade 2	6,893	96.04%	444	6.71%	1,672	25.26%
CRP-US grade 3	9,155	96.24%	701	7.96%	2,649	30.06%
CRP-US grade 4	11,093	95.57%	875	8.25%	3,537	33.36%
CRP-US grade 5	11,642	96.22%	961	8.58%	3,974	35.48%
CRP-US grade 6	13,843	95.45%	1,163	8.80%	4,934	37.34%
VxGL	126,410	82.80%	15,218	14.54%	61,230	58.50%
d-ELP	9,960	99.97%	452	4.54%	3,525	35.40%

Results show that the BAWF achieves broad coverage across these resources, ranging from 80.13% of COCA juvenile fiction word types, 82.80% of VxGL word types, and 83.50% of CPB-Lex word types, to 94.79%–97.45% of the CYP-Lex corpora, 95.45%–97.23% of the CRP-US graded reading corpora, and 99.97% of d-ELP word types. Given this high level of coverage, the BAWF provides a useful basis for estimating the morphological characteristics of words in children’s print and developmental vocabulary resources.

The results reveal clear differences in the estimated morphological complexity of print and instructional vocabulary. In CPB-Lex, 29.91% of BAWF-covered word types were classified as morphologically complex (one or more derivations or a compound), and 6.76% were classified as compounds. COCA juvenile fiction showed a higher level of complexity than CPB-Lex, with 37.57% of BAWF-covered word types classified as complex and 9.12% as compounds. In the CYP-Lex corpora, the proportion of complex word types increased from 42.27% in CYP-Lex 7–9 to 43.12% in CYP-Lex 10–12 and 45.80% in CYP-Lex 13+, while compounds accounted for approximately 10%–11% of covered word types across the three CYP-Lex datasets.

The CRP-US graded reading data showed a developmental pattern. In Grade 1, 17.49% of BAWF-covered word types were morphologically complex and 5.46% were compounds. By Grade 6, these estimates had increased to 37.34% complex word types and 8.80% compounds. Thus, across the elementary grades, the proportion of morphologically complex word types in graded reading materials approximately doubled, and the proportion of compounds also increased steadily.

The d-ELP showed a level of complexity similar to the upper elementary CRP-US grades, with 35.40% of BAWF-covered word types classified as complex and 4.54% as compounds. The VxGL list showed the highest estimated morphological complexity overall: 58.50% of BAWF-covered word types were complex, and 14.54% were compounds. This likely reflects the tendency of instructional and grade-level vocabulary resources to include a relatively high proportion of derived, compound, and otherwise morphologically complex words.

Morpheme count also correlated with both grade-level placement and word-reading difficulty: words assigned to higher grades in the VxGL contained more morphemes on average, *r* = 0.275, *p* < 0.001, *N* = 104,672, and words rated as more difficult in the d-ELP also contained more morphemes, *r* = 0.270, *p* < 0.001, *N* = 9,957. Morpheme count was also positively associated with d-ELP grade-equivalent scores, *r* = 0.233, *p* < 0.001, *N* = 9,957. Together, these patterns show that morphological complexity is not distributed evenly across the vocabulary children encounter, but accumulates as texts and instructional expectations become more demanding.

We examined coverage of school print by the curated set of word families with familiarity ratings above 3.5, indicative of approximately 90% of people knowing these words (Brysbaert et al., 2025). In addition to examining CPB-Lex and CYPLex, we added the Juvenile Fiction section of the Corpus of Contemporary American English (Davies, 2008) and a lexicon of U.S. core reading programs from Grades 1–6 (henceforth CRP-US) from Graves et al. (2019). We estimated how many familiar word families would be required to reach 90% and 95% coverage of the words (tokens) in school-aged texts. Excluding compounds, words not found in Wiktionary, proper nouns (e.g., character names) and abbreviations from the corpora, we estimated that learning approximately 2,100–3,100 familiar word families was sufficient to reach 90% token coverage across the school-aged corpora examined. Reaching 95% token coverage required a larger but still reasonable 4,100–7,900 familiar word families, depending on the corpus. These findings suggest that a relatively compact set of familiar word families can provide broad lexical access to school-aged print.

### RQ2: How much of this vocabulary is estimated to contain Greek-Latin roots?

Table 5 reports the estimated prevalence of words with classical roots across children’s print environments. These estimates include compound words in which at least one root-morpheme has been identified as classical in the BAWF. The results show that Greek-Latin root morphology plays a substantial role in the word types children encounter. Approximately 20% of unique word types in picturebooks, represented by CPB-Lex, are estimated to contain classical roots. This proportion rises to approximately 29% for ages 7–9, and 30% for ages 13+. COCA juvenile fiction shows a somewhat lower type estimate, at 23%. The CRP-US graded reading data show a clear developmental increase in the proportion of word types with classical roots. In Grade 1, 19% of word types are estimated to contain Greek-Latin roots. By Grade 6, this rises to 30%. Thus, across both UK and US children’s reading materials, classical root morphology becomes increasingly prominent in the vocabulary available to older readers.

**TABLE 5:** Estimated Greek-Latin root morphology in children’s print environments.

Database	Word types with classical roots	Type %	Word tokens with classical roots	Token %
CPB-Lex	5158	20.18	68986	6.16
CYP-Lex 7–9	15385	29.11	860568	7.71
CYP-Lex 10–12	20552	28.97	1714735	7.86
CYP-Lex 13+	27497	30.22	3153719	8.46
COCA Juv_Fic	12238	23.08	242774	8.28
CRP-US grade 1	606	19.75	6205	9.70
CRP-US grade 2	1595	23.14	35337	13.47
CRP-US grade 3	2347	25.64	49349	13.98
CRP-US grade 4	3110	28.04	63161	14.75
CRP-US grade 5	3420	29.38	70118	14.82
CRP-US grade 6	4226	30.53	77749	14.64

Examples of the word types with such roots that children are exposed to include *excited* (cit_citare, “to rouse/set in motion”); *captain* (capit_caput, “head”) *incredible* (cred_credo, “to believe”), *perfect* (fect_fact_facere, “to make/do”), *sound* (son_sonus, “sound”), and special (spec_spect_specere, “to look at”). With regard to token frequency, Greek-Latin root words were estimated to be ∼6% of running words in CPB-Lex, rising to ∼8% in CYP-Lex 7–9 and CYP-Lex 10–12, and about ∼8.5% in CYP-Lex 13+. COCA juvenile fiction shows a highly similar token estimate of ∼8.2%. The CRP-US graded reading data show even higher token coverage, increasing from ∼13% in Grade 1 to approximately 15% by Grades 4–6.

A few caveats need to contextualize the results. First, token frequency can be influenced by repeated occurrences of a relatively small number of frequent root-bearing words, and root assignments may be etymologically/pedagogically debatable or error. Secondly, the BAWF estimates are based on Wikipedia/Wiktionary-derived information and may not capture every possible Greek-Latin etymological root in the database’s wordforms. Some of the Wikipedia list of classical roots may also be considered by some linguists to be bound combining forms (e.g., *con-, ab-).* Thirdly, the prevalence of words in these print environments tagged in the BAWF as having Greek-Latin roots reflects only exposure patterns, not children’s awareness of those roots as meaning-bearing units.

Nonetheless, the results indicate that Greek-Latin roots are not rare in children’s print experience. The BAWF supports the instructional selection of these words to help children become aware of them as meaning-bearing units. Table 6 reports the 100 most productive classical roots in the BAWF (V1). Word families are ranked by the number of wordforms in each family. These 100 roots and their allomorphs contribute to ∼38,983 wordforms in the BAWF (excluding compound words), a good return on a small number of bound morphemes. As Table 6 shows, these roots are many of the familiar ones in K–12 teaching materials, such as *spec(t), ject, tract, struct* etc.

**TABLE 6:** The 100 most productive Greek-Latin word roots.

Morpheme (1–50)	Meaning	Morpheme (50–100)	Meaning
fac/fact/fect/fic	do/make	line	line
st/sta	stand/cause to stand	claud/clud/claus/clus	close/shut
con/co/col/com/cor	with/together	cur	care/cure
leg/lect	choose/gather/read	gnosc/gnit	know
ad/a/ac/af/ag/al/am/an/ap/ar/as/at	to/toward; addition	grad/gred/gress	walk/step/go
gen/gon	birth/beget/race/kind	rot	wheel/turn
ag/ig/act	do/go/move	loc	place
spec/spic/spect	look/see	quer/quir/quesit/quisit	search/seek
mov/mot/mut	move/motion	fund/fus	pour
pon/posit	put/place	trah/tract	draw/pull
form	shape/form	caed/cid/caes/cis	cut/kill
vers/vert	turn	ser	silk/join/bind
vid/vis	see	spir	breathe
nasc/nat	born	flu/fluv/flux	flow
prem/prim/press	press	phan/phen	show/visible
cap/cip/capt/cept	hold/take	frag/frang/fring/fract	break
ced/cess	move/yield/go	can/cin/cant/cent	sing
mit/miss	send	graph	write/draw
port	carry	pell/puls	drive/push
mod	measure/change	voc	call/voice
jac/ject	cast/throw	gramm	letter/writing
part	part	meter/metr	measure
ab/a/abs/au	away from/down/off	fig/fing/fict	form/shape
de	down/away/removing	ordin	order
rect/reg/rig	straight/direct/rule	ger/gest	bear/carry
col/cult	cultivate/till/inhabit	prehend/prend/prehens	grasp
log/-logy	word/reason/speech	prob	worthy/good/prove
plic	fold	ten/ton	stretch/tone
fer	bear/carry	min	small/less
fin	end	ple/plet	fill
duc/duct	lead	fa	say/speak
pro	before/in front of	soci	companion/group
plan	flat/plane; wander	cad/cid/cas	fall
erg/org/urg	work	put	prune/reckon/think
tend/tens	stretch/strain	sex	six/sexual
hab/hib/habit/hibit	have/hold	chro/chrom	color
ven/vent	come	pet	strive toward/seek
sent/sens	feel/sense	tribu	pay/assign
capit/cipit	head	pot	power/able
serv	save/protect/serve	sec/sect/seg	cut
par	equal/prepare	ut/us	use
val	strength/worth	flect/flex	bend
man/manu	hand	mer	part/portion
scrib/script	write	ten/tin/tent/tain/tinu	hold/keep
bal/bel/bol	throw	arch/arche/archi	chief/first/rule
sign	sign/mark	dic/dict	say/speak/proclaim
pend/pens	hang/weigh/pay	pati/pass	suffer/feel/endure
stru/struct	build/make up	ped	foot
volv/volut	roll	the	place/put
div/diff	different/divide	ly/lysi/lyt	loosen/break down

We conducted analyses to see ranking differences across children’s print databases. Overall, the rank-order patterns were broadly similar: e.g., *spec(t)/spic* frequently amongst the top-ranked families in words relatively transparent such as *aspect, expect, inspect, respect*, and *perspective*. Similarly, *mov/mot* was common, including *move, motion*, and *emotion*. There were differences, however: e.g., *vid/vis* was the second most productive root in CRP-US Grade 1 (*view, visual, visit)* but not always in other corpora, which may relate to the increasing size of the corpora/number of words in the reading programs at each grade. The BAWF helps us narrow down which root families are productive in specific children’s corpora.

### RQ3: What are the most productive prefixes and suffixes, and what is their lexical coverage?

The BAWF contains 1,016 distinct prefix entries and ∼69,286 wordforms with at least one prefix. When we use the Wiktionary-listed prefixes only and group allomorphs together (i.e., the column Prefix_1_All_Forms), the number is 583 distinct prefix entries. Table 7 lists the 100 most productive prefixes and suffixes in the BAWF, ranked by productivity (the number of word forms in which each appears). The 100 most productive prefix entries cover ∼65,476 of these prefixed wordforms, or ∼94%. A relatively small number of highly productive prefixes accounts for the overwhelming majority of prefixed wordforms.

**TABLE 7:** The 100 most productive prefixes and suffixes (ranked by productivity).

Prefixes				Suffixes			
1–25	26–50	51–75	76–100	1–25	26–50	51–75	76–100
un-	post-	down-	pan-	-er	-ful	-ery	-ide
re-	poly-	neo-	sym-	-ion	-ance	-hood	-ol
non-	mono-	epi-	after-	-al	-en	-ling	-t
de-	be-	macro-	hemi-	-y	-ar	-o	-ose
co-	counter-	peri-	paleo-	-ize	-n	-esque	-oma
in-	di-	endo-	apo-	-ly	-ie	-some	-wise
over-	up-	homo-	allo-	-ate	-ise	-ase	-it
pre-	semi-	extra-	ana-	-ic	-ite	-sis	-ry
a-	tri-	hetero-	meso-	-able	-like	-ster	-one
dis-	e-	syn-	penta-	-ness	-ee	-id	-itize
sub-	bi-	tele-	hexa-	-ist	-an	-ette	-emia
im-	meta-	dys-	sur-	-ity	-in	-ium	-arian
anti-	intra-	exo-	sy-	-ive	-age	-itis	-st
micro-	nano-	uni-	em-	-ism	-ship	-osis	-ty
super-	per-	eu-	equi-	-less	-oid	-ward	-gen
inter-	mega-	retro-	contra-	-or	-eth	-yl	-ane
ex-	auto-	ob-	off-	-ian	-ous	-et	-us
under-	fore-	proto-	arch-	-ant	-ure	-on	-cy
en-	hypo-	mal-	on-	-ment	-ine	-eer	-ile
multi-	mid-	su-	kilo-	-ed	-let	-ancy	-d
pro-	para-	tetra-	ortho-	-est	-ess	-ary	-ene
mis-	pseudo-	ab-	sus-	-ify	-ia	-r	-i
out-	mini-	supra-	milli-	-ious	-th	-acy	-ectomy
trans-	ultra-	circum-	back-	-ory	-le	-man	-ome
hyper-	iso-	dia-	cyber-	-ish	-dom	-ese	-etic

For suffixes, the BAWF contains 1,125 distinct raw suffix entries and 94,448 wordforms with at least one suffix. The 100 most productive suffixes cover ∼89,888 of these suffixed wordforms, or ∼95%. Unlike the prefix analysis, Wiktionary allomorph-grouped suffix data were not available in the present version of the BAWF, so suffix estimates are based on the raw GPT-coded suffix labels. Some of these entries likely represent allomorphic variants.

Table 8 shows the percentage of BAWF-covered word types in each database that contain one of the top 100 most productive prefixes or suffixes in the BAWF, excluding compounds. It also shows how many word types contain rarer prefixes or suffixes outside the top 100 list, and the proportion of word types that are both prefixed and suffixed, only prefixed, or only suffixed.

**TABLE 8:** Patterns of affixation in print vocabulary databases (% word types covered).

Database	With top 100 prefix	With top 100 suffix	Prefixed not top 100	Suffixed not top 100
CPB-Lex	8.73%	22.62%	0.50%	0.96%
CYP-Lex 7–9	14.57%	29.56%	0.66%	1.10%
CYP-Lex 10–12	15.30%	29.81%	0.73%	1.14%
CYP-Lex 13+	17.28%	31.89%	0.74%	1.23%
COCA Juv_Fic	12.99%	26.20%	0.61%	1.00%
CRP-US grade 1	5.85%	12.34%	0.67%	0.92%
CRP-US grade 2	8.50%	19.08%	0.66%	1.30%
CRP-US grade 3	10.21%	22.39%	0.70%	1.25%
CRP-US grade 4	11.76%	24.75%	0.87%	1.47%
CRP-US grade 5	12.71%	26.25%	0.80%	1.43%
CRP-US grade 6	13.82%	27.71%	1.00%	1.60%
VxGL	24.05%	41.30%	1.57%	2.09%
d-ELP	13.88%	31.11%	0.69%	0.88%
	**Both prefixed and suffixed**	**Only prefixed**	**Only suffixed**	
CPB-Lex	2.98%	6.25%	20.47%	
CYP-Lex 7–9	5.93%	9.30%	24.57%	
CYP-Lex 10–12	6.50%	9.52%	24.27%	
CYP-Lex 13+	7.76%	10.25%	25.15%	
COCA Juv_Fic	5.15%	8.45%	21.92%	
CRP-US grade 1	1.60%	4.93%	11.56%	
CRP-US grade 2	3.01%	6.15%	17.26%	
CRP-US grade 3	4.16%	6.76%	19.40%	
CRP-US grade 4	5.13%	7.51%	20.92%	
CRP-US grade 5	5.46%	8.05%	22.03%	
CRP-US grade 6	6.38%	8.44%	22.77%	
VxGL	12.77%	12.85%	30.31%	
d-ELP	5.24%	9.33%	26.75%	

Table 8 shows a consistent dominance of suffixation over prefixation across the lexical databases. In every corpus, the proportion of word types containing at least one of the top 100 suffixes was substantially higher than the proportion containing one of the top 100 prefixes. In CPB-Lex, 8.73% of BAWF-covered word types contained a top-100 prefix, whereas 22.62% contained a top-100 suffix. This pattern was also evident across the CYP-Lex corpora: top-100 prefix coverage increased from 14.57% in CYP-Lex 7–9 to 17.28% in CYP-Lex 13+, while top-100 suffix coverage remained higher, ranging from 29.56% to 31.89%. COCA juvenile fiction showed a similar pattern, with 12.99% of covered word types containing a top-100 prefix and 26.20% containing a top-100 suffix.

The CRP-US graded reading data showed a clear developmental increase in affixed vocabulary. Top-100 prefix coverage rose from 5.85% in Grade 1 to 13.82% in Grade 6, while top-100 suffix coverage increased from 12.34% to 27.71%. Words containing both a prefix and a suffix also increased across the grades, from 1.60% in Grade 1 to 6.38% in Grade 6. A similar age-related pattern was observed in CYP-Lex, where both-prefixed-and-suffixed word types increased from 5.93% in CYP-Lex 7–9 to 7.76% in CYP-Lex 13+. The highest type-based estimates were observed in VxGL, where 24.05% of covered word types contained a top-100 prefix, 41.30% contained a top-100 suffix, and 12.77% contained both a prefix and a suffix. Words using non-top-100 affixes were rare. Across datasets, polymorphemic words not affixed with these top 100 morphemes were extremely uncommon.

In the children’s print data, examples of words that can be identified using the BAWF carrying top-100 prefixes included relatively transparent forms such as *unhappy, unable, nonfiction, preview, reread, rewrite, disappear*, and *misunderstand*. Examples of words carrying top-100 suffixes included tea*cher, reader, action, selection, national, quickly, kindness, happiness, readable, and movement*. However, not all prefixes/suffixes need to be instructional targets, and not all words containing them are equally semantically transparent. Some lower-ranked affixes in Table 7, such as *hemi-, paleo-, -osis, and -esque*, may be more relevant for older learners and disciplinary literacy contexts. Nevertheless, many of the high-frequency affixes appear quite useful. Overall, the results show that exposure to such affixation patterns becomes more common across the school years.

## Discussion

The three research questions guiding this study investigated estimates of the morphological complexity of print environments across the school years, the productivity of English affixes, and patterns of classical roots. We also reported on the scope and accuracy of the BAWF relative to existing morphological databases. The results converge on a coherent account of English morphological structure as it bears on children’s reading development. Each is taken up in turn below, followed by a discussion of limitations and directions for future research.

### Morphological complexity in children’s print environments

By applying the BAWF-based analysis to the recently developed lexical databases of children’s print environments, we found a pronounced and systematic increase in morphological complexity with grade and age. The contrast between CPB-Lex, where 29.91% of BAWF-covered picturebook word types were morphologically complex, and the CYP-Lex databases, where complexity increased from 42.27% in CYP-Lex 7–9 to 45.80% in CYP-Lex 13+, underscores how substantially the morphological demands of reading change across the school years.

These results are similar to the estimates reported by Korochkina and Rastle (2025) using MorphoLex. The developmental pattern was also evident in the US core reading programs (Graves et al., 2019): morphologically complex word types increased from 17.49% in Grade 1 to 37.34% in Grade 6, while compounds increased from 5.46% to 8.80%. The VxGL pattern of a high rate of polymorphemic words likely reflects the intentional inclusion of derivationally rich vocabulary in instructional lists. The d-ELP contained 35.40% morphologically complex word types.

The patterns of Greek–Latin root morphology warrants particular attention. Approximately 20% of unique word types in picturebooks contain a classical root, rising to approximately 30% in the CYP-Lex corpora for readers aged 7–13+. COCA juvenile fiction showed a somewhat lower type estimate, at 23%, but a similar token estimate to the CYP-Lex corpora. Importantly, the CRP-US graded reading materials again showed a clear developmental increase: Greek–Latin-root word types rose from ∼19% in Grade 1 to ∼30% in Grade 6.

These estimates suggest that classical root morphology is not a feature of advanced vocabulary alone, but common in the texts children encounter throughout schooling. Teaching a carefully selected set of transparent and productive classical roots may therefore support children’s morphological awareness and help them notice meaningful word parts. The BAWF can assist teachers, researchers, and curriculum developers in identifying such words.

### Affix productivity and the concentration of morphological structure

The study demonstrates that English affixation is organized around a small set of highly productive forms. In the BAWF, the raw GPT parses yielded 1,016 distinct prefix entries and 1,125 distinct suffix entries. Interestingly, the top 100 prefixes accounted for ∼94% of preficed wordforms, and the top 100 suffixes accounted for ∼95% of suffixed wordforms. This concentration is consistent with the “tremendous productivity of language” described by Brysbaert et al. (2016), p. 4) and with Nagy and Anderson’s (1984) observation that morphological knowledge has substantial leverage for vocabulary acquisition. The present findings extend that conclusion using a much larger lexical database: a relatively small set of high-yield affixes captures the overwhelming majority of prefixed and suffixed wordforms in the BAWF.

The dominance of suffixation over prefixation across all corpus comparisons (Table 8) is similar to previous findings (e.g., Dawson et al., 2023) for the Oxford Children’s Reading Corpus), and extends it by adding prefix data and covering a larger range of corpora. Across all corpora, words containing one of the top 100 suffixes were substantially more common than words containing one of the top 100 prefixes. This suggest that suffixation may be an instructional priority.

The CRP-US graded reading data strengthen the developmental interpretation. In these core reading program materials, top-100 prefix coverage rose from 5.85% in Grade 1 to 13.82% in Grade 6, while top-100 suffix coverage increased from 12.34% to 27.71%. Words containing both a prefix and a suffix also increased across grades, from 1.60% in Grade 1 to 6.38% in Grade 6. Thus, the increasing morphological demands of school reading are visible in the graded materials children encounter in classroom reading programs.

Words containing affixes outside the top 100 were rare. This pattern suggests that the morphological structure of children’s print is highly concentrated: a small inventory of productive prefixes and suffixes accounts for most affixed vocabulary.

### Implications for reading and literacy development

These findings have clear literacy implications. If most affixed words in children’s print draw on a relatively small set of productive prefixes and suffixes, then morphology instruction can be systematic and efficient. Teaching high-yield affixes such as *un-, re-, non-, de-, -er, -ion, -al, -y*, and -*ize* may support children’s ability to decode, spell, and infer the meanings of large numbers of unfamiliar words. The developmental increase in prefixation, suffixation, and combined prefix-suffix structures further suggests that morphological instruction should not be delayed until secondary school; rather, it should be introduced early and expanded as children encounter increasingly morphologically complex texts.

The empirical picture that emerges across the three research questions has implications for how morphological complexity is investigated in future reading research. Most consequentially, the findings create the conditions for an updated and substantially more comprehensive version of the analysis conducted by Nagy and Anderson (1984), whose estimate of three to four new words derivable from each known base remains among the most widely cited claims in vocabulary research. That analysis was constrained by the resources available in the early 1980s: the Carroll et al. (1971) frequency dictionary covered Grades 3 through 9 as a matter of sampling necessity, not theoretical choice. The BAWF spans essentially the full contemporary English lexicon as attested in two major national corpora, is aligned to lexical databases representing children’s print environments from kindergarten through college, and carries frequency information at both word-form and family levels. The principal analytic element still needed before such an update could be undertaken is a semantic transparency rating for derived forms - the criterion Nagy and Anderson used to determine whether a derived word could be counted as inferable from its base or should be treated as a separate vocabulary item. That component cannot be extracted from the existing BAWF and would require new data collection; it represents the most important methodological next step for this line of research.

For instructional research, the findings bear on questions of sequence and priority in morphology instruction. The concentration of morphological structure in a small number of high-productivity affixes and classical roots means that prioritizing those forms maximizes lexical coverage per unit of instruction. A child who learns the prefix *un-* or the suffix *-tion* has access to a large portion of the morphologically complex words she will encounter; a child who learns only rare or low-productivity forms does not. These considerations do not themselves determine instructional sequence - questions of developmental readiness, cross-morphemic interactions, and instructional time remain - but they provide an empirical basis for conversations that have until now proceeded largely without large-scale lexical evidence. The finding that Greek–Latin roots are already present in picture books similarly suggests that classical morphology should not be treated as exclusively an upper-elementary concern; exposure to the most frequent roots may be valuable earlier than is commonly assumed (Crosson et al., 2025).

### Limitations and future research in the BAWF project

Word-list and database creation are ambitious, long-term endeavors that require years of iterative development, as exemplified by large-scale resources such as the BNC-COCA word family lists (Nation, 2017). The BAWF project is an evolving, open-access resource. While the database provides substantial coverage and enables new large-scale morphological analyses, it is not definitive and error-free. We have presented here estimates subject to signal-to-noise considerations. The BAWF (V1) was generated using a fine-tuned GPT-4 model trained on MorphoLex and augmented with Wiktionary-derived data, without exhaustive *post-hoc* human correction. There is much useful information across this vast database, but users will inevitably encounter parses that are incorrect, incomplete, or suboptimal.

Future development of the BAWF project will focus at least these key areas:

1. Enhanced Online Interface: Currently, many analytical tasks require downloading the raw database and executing code. We plan to build features allowing users to analyse custom text collections, so users will be able to evaluate the patterns of their own instructional materials.

2. Morphological Transparency Norms: The BAWF project is currently exploring crowdsourcing and fine-tuning LLMs in order to generate morphological transparency ratings for polymorphemic words. This will help distinguish between words that are more/less instructionally transparent.

3. Parse Refinement: Following resource development models that currently exist, the BAWF will be improved over time. There are limits on what can be done in any one project phase, and as researchers we needed to be mindful with publicly funded research (financial, human resource, environmental costs of LLMs). As previous large-scale word-list projects have shown (Nation, 2017), word family refinement of large lists comes with diminishing returns for increasingly small improvements. This does not mean they are unimportant, but they cannot all be completed at once. The open-access nature of the project will allow researchers, educators, and developers to create derived products or specialized versions of BAWF. In addition, the platform includes built-in systems for parse refinement and corrections. Future LLM models are likely to improve parses, and may allow for parses aligned with particular psycholinguistic theories.

## Conclusion

This study has reported the development and initial application of the British and American Word Families database, a morphological resource covering approximately 378,000 words of contemporary English with accuracy comparable to human-constructed databases and a corpus scope that substantially exceeds any existing alternative. Applied to four recently developed lexical databases representing children’s print environments, the BAWF reveals a consistent picture: morphological complexity is not uniformly distributed across the vocabulary children encounter but accumulates across the school years, and is dominated by a small set of highly productive affixes. Further Greek–Latin root morphology is present even in the earliest print environments. These findings bear directly on theoretical accounts of vocabulary development and on the questions that motivate intervention research in morphological instruction. The BAWF (V1) is released under a Creative Commons license, and the authors invite correction, extension, and reuse by the research community.

## Data Availability

This project used publicly available corpora. Further, all code and wordlists developed in this project are available in the Open Science Framework: https://tinyurl.com/43e3te39.

## Footnotes

1
https://cgg-projects.github.io/BAWF_V1/

2
https://www.wiktionary.org/

3
https://kaikki.org/

4
https://openai.com/

5
https://en.wikipedia.org/wiki/List_of_Greek_and_Latin_roots_in_English

6
https://en.wiktionary.org/wiki/Appendix:English_prefixes

7
https://en.wiktionary.org/wiki/Category:English_suffixes

8
https://en.wiktionary.org/wiki/Category:English_terms_by_prefix

9
https://en.wiktionary.org/wiki/Category:English_terms_by_suffix

10
https://tinyurl.com/53ya4sks
